# Knowledge Domain and Emerging Trends in Organic Photovoltaic Technology: A Scientometric Review Based on CiteSpace Analysis

**DOI:** 10.3389/fchem.2017.00067

**Published:** 2017-09-15

**Authors:** Fengjun Xiao, Chengzhi Li, Jiangman Sun, Lianjie Zhang

**Affiliations:** ^1^School of Humanities and Social Sciences, Beihang University Beijing, China; ^2^Hangzhou Dianzi University Hangzhou, China; ^3^Beijing Institute of Nanoenergy and Nanosystems, Chinese Academy of Sciences Beijing, China; ^4^Institute of Polymer Optoelectronic Materials and Devices, State Key Laboratory of Luminescent Materials and Devices, South China University of Technology Guangzhou, China

**Keywords:** organic photovoltaics, scientometrics, citespace, visualization analysis, emerging trends

## Abstract

To study the rapid growth of research on organic photovoltaic (OPV) technology, development trends in the relevant research are analyzed based on CiteSpace software of text mining and visualization in scientific literature. By this analytical method, the outputs and cooperation of authors, the hot research topics, the vital references and the development trend of OPV are identified and visualized. Different from the traditional review articles by the experts on OPV, this work provides a new method of visualizing information about the development of the OPV technology research over the past decade quantitatively.

## Introduction

For the requirement of new and renewable source of energy in today's world, photovoltaic (PV) technology which can convert solar energy to electricity have attracted scientists' great interests. Although, the development of photovoltaic (PV) technology based on inorganic materials are dominating the market at present (Green et al., 2015), the widespread application of PV technology is limited by the high cost of production and related environmental problems. Organic photovoltaic (OPV) technology is developing fast in recent years due to its unique advantages, such as, synthetic variability of materials, (Liu et al., 2015) the possibility of producing lightweight, flexible, easily processed, and inexpensive solar cells and environmental sustainability (Kaltenbrunner et al., 2012; Sondergaard et al., 2012; Sun et al., 2012; Singh and Kushwaha, 2013; Chen K. S. et al., 2014; Green et al., 2015). So it is a promising technology which can be used for fabricating thin-film solar cells.

The power conversion efficiency (PCE) of OPV has been improved from 1% to over 12%, particularly through the efforts of the last decade (Dou et al., 2013; Jung et al., 2016; Li et al., 2016; Green et al., 2017; Singh and Kushwaha, 2017; Zhao et al., 2017). The main developments of OPV involve in the following aspects: designing and synthesizing new conjugated polymer materials, understanding and controlling the film morphology, illuminating the device mechanisms, constructing new device architectures. All of these achievements promote the rapid progress of the OPV technology. Therefore, the OPV technology is presented as an exciting research field, which attracts a huge amount of researchers involved in chemistry, material science, physics, and engineering. It is meaningful to visualize the knowledge domain of OPV, which will be helpful to explore the research, the development history as well as the future trends clearly.

This paper focuses on the network of co-authors, co-occurring keywords, co-citation reference and the burst of the co-citation reference resulted from CiteSpace which is a visualization tool to analyze the references obtained from the Web of Science Core Collection (Lee et al., 2016). So, the knowledge domains, quantified research patterns and trends about OPV can be explored, which is helpful to obtain more accurate and complete information of the OPV research field.

## Method

### Data collection

The data used for bibliometric analysis was collected from the Web of Science (WoS) Core Collection of Thomson Reuters including SCI-Expanded, SSCI, A&HCl, CPCI-S, CPCI-SSH, ESCI, CCR-Expanded and IC. The first article about OPV was published by Garnier et al. (Horowitz et al., 1984). Thus, the timespan for search was from 1984 to 2016. The topic search consists of index words about organic photovoltaics (OPV) as follows: “organic solar cells or polymer solar cells or small molecule solar cells.” This search resulted in 40,069 records and 35,231 records with a document type of article included. The article document type records were exported to CiteSpace for the further analysis (Chen, 2006). While the most recent article document type records of 2,795 were also collected on the date of 07/11/2017 with a timespan from 2017 to 2017. These documents can be used to study the nearest development trend of OPV.

### CiteSpace

CiteSpace is a Java application for analyzing and visualizing co-citation networks (Chen, 2004), including co-citation references, co-authors, and co-occurring keywords, (Chen, 2013) which facilitates to deliver the results of OPV knowledge domain. CiteSpace is related to three central concepts: burst detection, betweenness centrality, and heterogeneous networks. Three practical issues, identifying the nature of a research front, labeling a specialty and detecting emerging trends and abrupt changes in a timely manner, could be addressed by these concepts (Chen, 2006). And the procedural steps required in CiteSpace are as follows: time slicing, thresholding, modeling, pruning, merging, and mapping. While pruning, which is a potentially valuable option when dealing with a dense network, is not always necessary (Chen, 2004). The primary source of input data for CiteSpace is the Web of Science.

After the visualization of input date through CiteSpace, we can explore the knowledge domains in a specific topic. Burst detection algorithm can be adapted for detecting sharp increases of interest in a specialty (Kleinberg, 2002). In CiteSpace, a current research front is identified based on such burst terms extracted from titles, abstracts, descriptors, and identifiers of bibliographic records. CiteSpace also makes it easier for users to identify pivotal points by recognizing the nodes with high betweenness centrality (Freeman, 1978). Pivotal points are highlighted in the display with a purple ring in order to stand out in a visualized network (Chen, 2006).

The betweenness centrality is defined in the following Equation (1).

(1) $$Centrality\left(node_{i}\right)=\sum_{i\neq j\neq k}\frac{\rho_{jk}\left(i\right)}{\rho_{jk}}$$

In the Equation (1), ρ_*jk*_ represents the number of shortest paths between node *j* and node *k*, and ρ_*jk*_(*i*) is the number of those paths that pass through *node*_*i*_. Additionally, in the weighting directed graph, the Equation (1) includes several types of transformation. At the document level, the importance of each document in a co-citing network can be partially evaluated by the indicator betweenness centrality (Li M. N. et al., 2017).

Therefore, in what follows, bibliometric analysis based on CiteSpace is utilized to explore the hidden patterns and reasons for the growth on OPV technology. In addition to a traditional review of literature by experts, a bibliometric analysis can reveal another facet of the research fronts on OPV by micro and quantitative means.

## Results and discussion

### Publication years and journals

**Figure 1:**
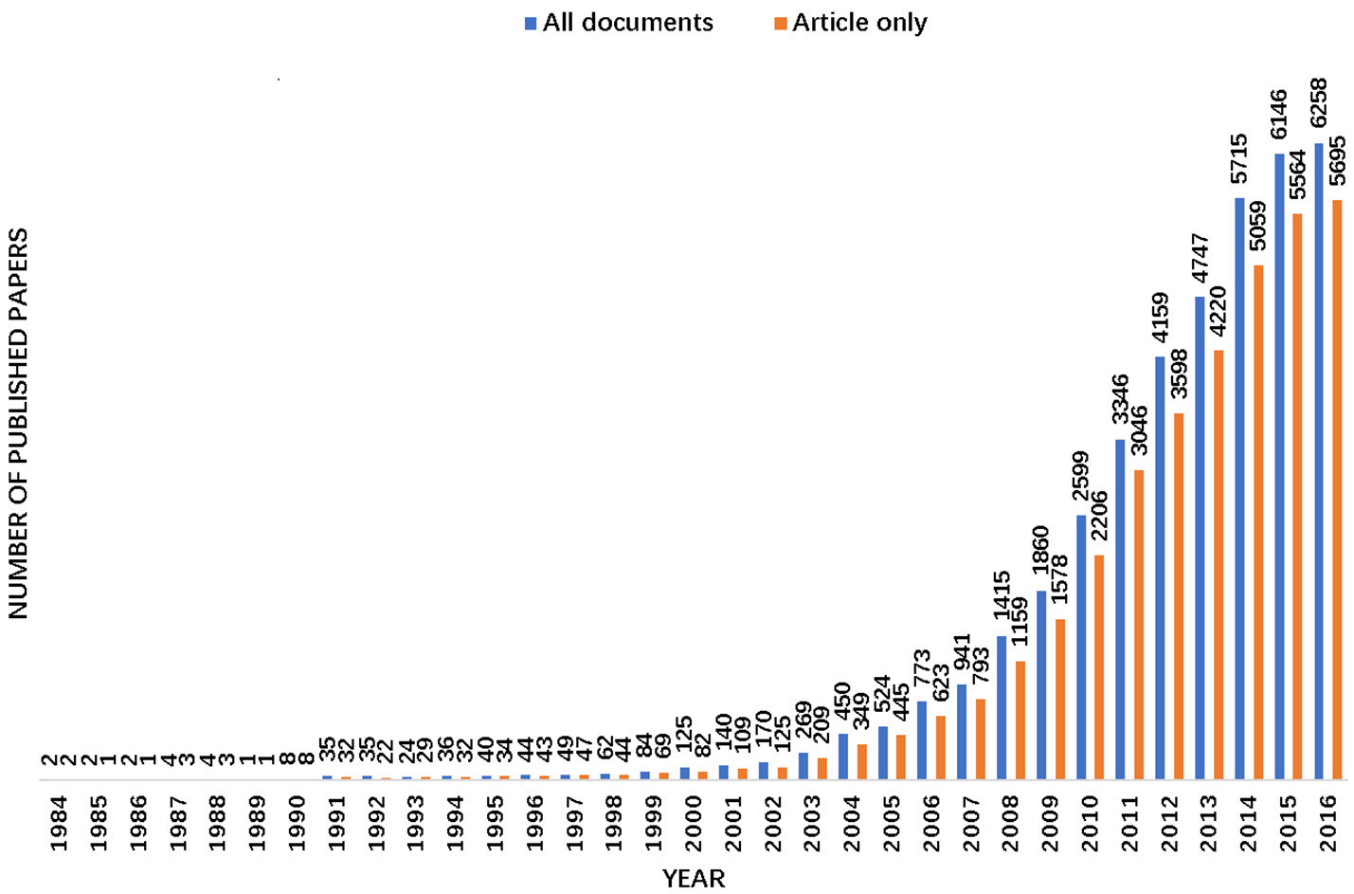
Time sequence of relevant published papers of all documents and articles on organic solar cells in WoS.

The first paper about OPV “Protection of normal-gaas photoanodes by photoelectronchemical grafting of poly (3,4-dimethyl-thiophene) films” was published in 1984 by Garnier et al. (Horowitz et al., 1984) which stands for the prototype of the OPV research field. After that the publications about OPV are growing persistently. The number of all types of published documents increased from 2 in 1984 to 6258 in 2016 as well as the number of published articles increased from 2 to 5695 as shown in Figure 1. A non-linear correlation of the number of published papers and the published year series data reveals that the growth pattern in Figure 1 is very close to the exponential function.

As shown in Figure 1, one might conclude that the number of relevant publications on OPV have increased rapidly since 2005. At that year, several important articles which stimulated the development of OPV were published, such as “High-efficiency solution processable polymer photovoltaic cells by self-organization of polymer blends” by Li et al. (2005) which focused on the polymer poly (3-hexylthiophene) and “Thermally stable, efficient polymer solar cells with nanoscale control of the interpenetrating network morphology” by Ma et al. (2005). These two highlighted articles together with others stimulated the development of OPV, as a result, various new materials spring up and the performance of OPV devices have been improved continuously as the efforts of researchers.

All the article records on OPV were distributed in 87 journals. Journal of Physical Chemistry C ranks first in the number of publications (1,477), followed by Solar Energy Materials and Solar Cells (1,425), and Applied Physics Letters (1,165). The top 10 most productive journals are presented in Table 1. All of this can provide important submission information for new researchers.

**Table 1:** The top 10 most productive journals.

**The name of journals**	**The number of published papers**
Journal of Physical Chemistry C	1,477
Solar Energy Materials and Solar Cells	1,425
Applied Physics Letters	1,165
Organic Electronics	1,142
ACS Applied Materials Interfaces	1,129
RSC Advances	1,005
Journal of Materials Chemistry A	911
Advanced Materials	802
Macromolecules	768
Synthetic Metals	756

### Co-authorship

Considering the volume of published documents, the most productive authors in OPV research were Y. F. Li with 405 articles, followed by J. H. Kim, M. Grätzel, Y. Yang, F. C. Krebs, J. Zhang, H. Kim, Y. Cao, C. J. Brabec and Y. Li. Then a collaboration network for the productive authors was analyzed by CiteSpace. A timespan from 2006 to 2016 with a time slice of 1 year was chosen for the analysis and the selection criteria was top 50% per-slice.

**Figure 2:**
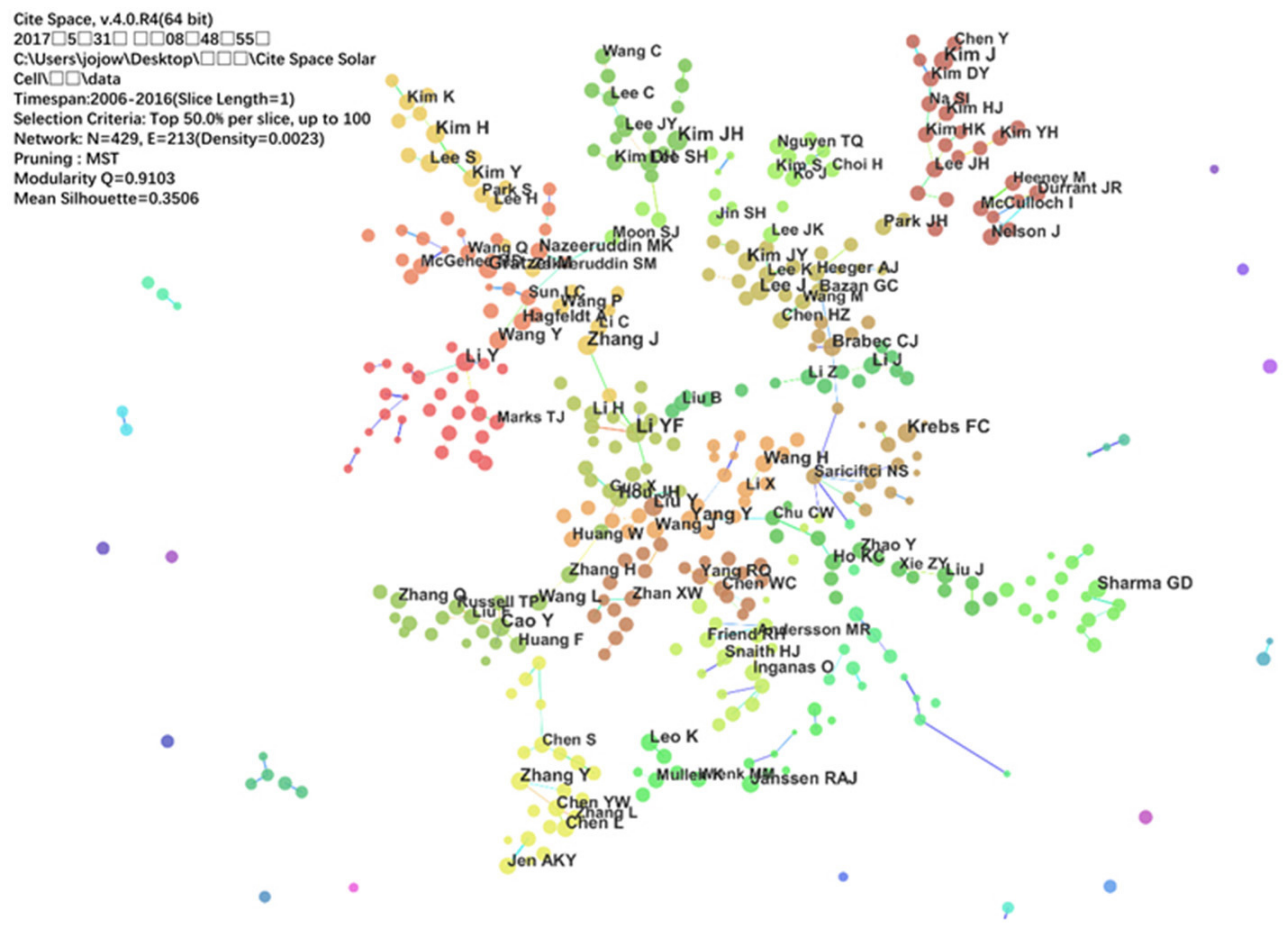
The cooperation network of productive authors.

The collaboration map is presented in Figure 2. The size of circles represents the amount of publications of the authors, and the shorter distance between two circles suggests the more collaboration between individual authors. The color of circles stands for the authors in the same cluster. It can be noticed that many authors tended to cooperate with a relatively stable group of the collaborators, generating several major clusters of authors, each of which usually have two or more core authors, for example, the cluster with Y. F. Li, the cluster with Y. Cao, the cluster with A. J. Heeger and G. C. Bazan and so on. The major clusters with core author showed in Figure 2 also present the most representative research groups in the field of OPV, which can offer highly individualized scientific research information to other researchers.

### Co-occurring keywords analysis

**Figure 3:**
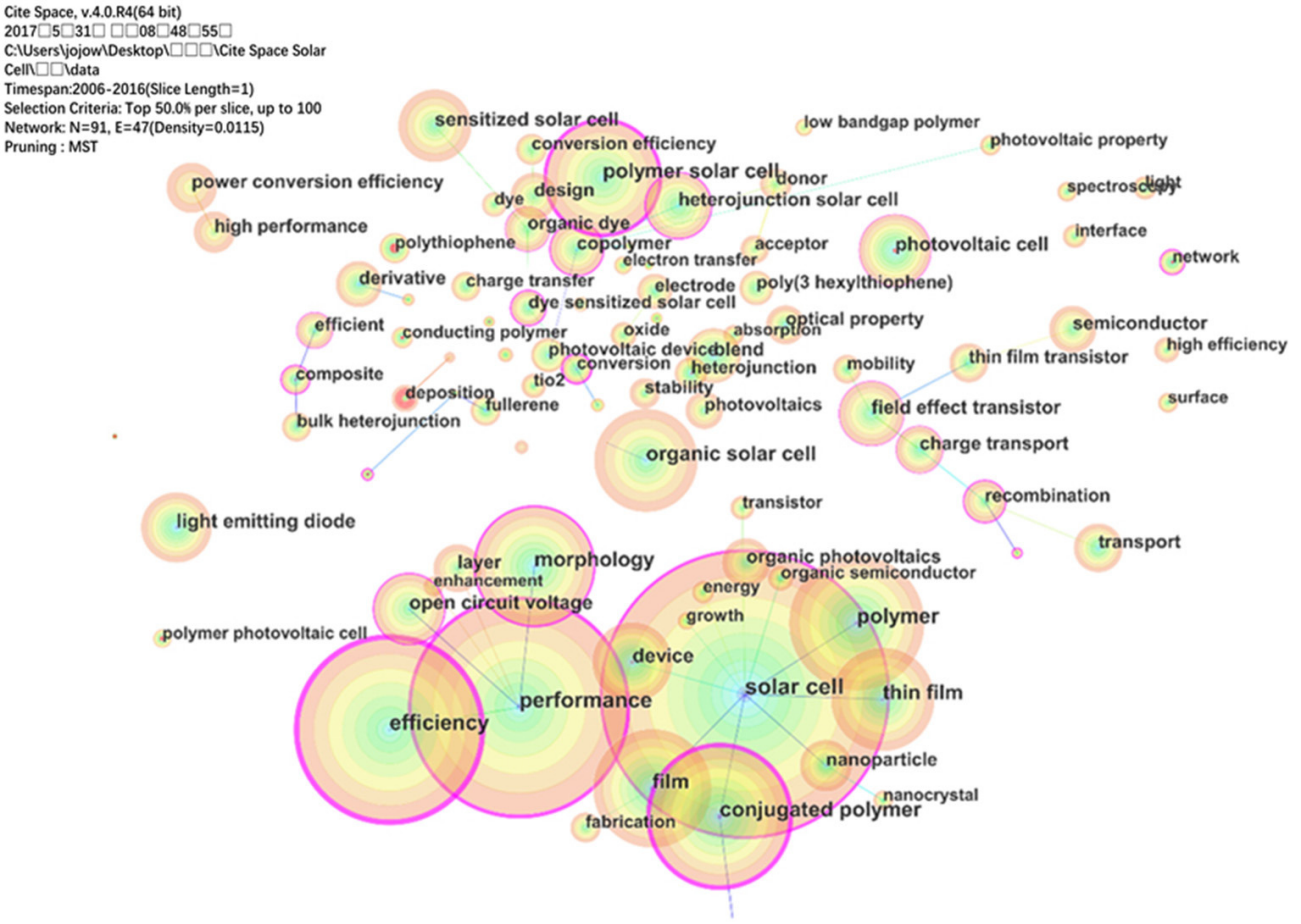
The keywords co-occurrence network.

The co-occurring keywords reflect research hotspots in OPV field. A timespan from 2006 to 2016 with a time slice of 1 was selected for the analysis and the top 50 most cited or occurred items from each slice was chosen. As shown in Figure 3, a simplified co-occurring keyword network was obtained with the minimum spanning tree (MST) algorithm. The nodes represent the keyword and the size of each node is corresponding to the co-occurring frequencies of keywords. The colors of co-occurring links among keywords indicate the temporal orders: oldest in blue, and newest in orange. “solar cell” was enabled with the largest frequency of 8998, followed by “performance” (6,823), “efficiency” (5,762) and “conjugated polymer” (4,427). Other commonly used words are “film” (3,823), “polymer solar cell” (3,612), “morphology” (3,771), “open circuit voltage” (2,232) and so on. Most of these nodes marked by purple circle indicate good centrality and the importance of these keywords. Among these keywords “efficiency” had the highest centrality (1.34), followed by “conjugated polymer” (1.19), “performance” (0.98), “polymer solar cell” (0.96). So, conjugated polymers, which were used as the active layer of OPV devices, were widely studied in OPV research filed.

Notably, the keywords such as “polythiophene,” “deposition,” “polymer photovoltaic cell,” and “network” were the nodes with a red inner ring, which indicated the frequency changed considerably. In other words, these nodes represent the emerging trends in OPV field with strongest burst. “network,” with burst strength of 37.8244, begin burst from 2006 to 2009; “polythiophene” (74.4284) begin burst from 2006 to 2011; “polymer photovoltaic cell” (28.2089) begin burst from 2006 to 2011; “deposition” (9.3794) begin burst from 2014 to 2016. As we know, “deposition” is a processed method related to perovskite solar cell which is the hottest topic solar cell technology recently.

### Document co-citation analysis

**Figure 4:**
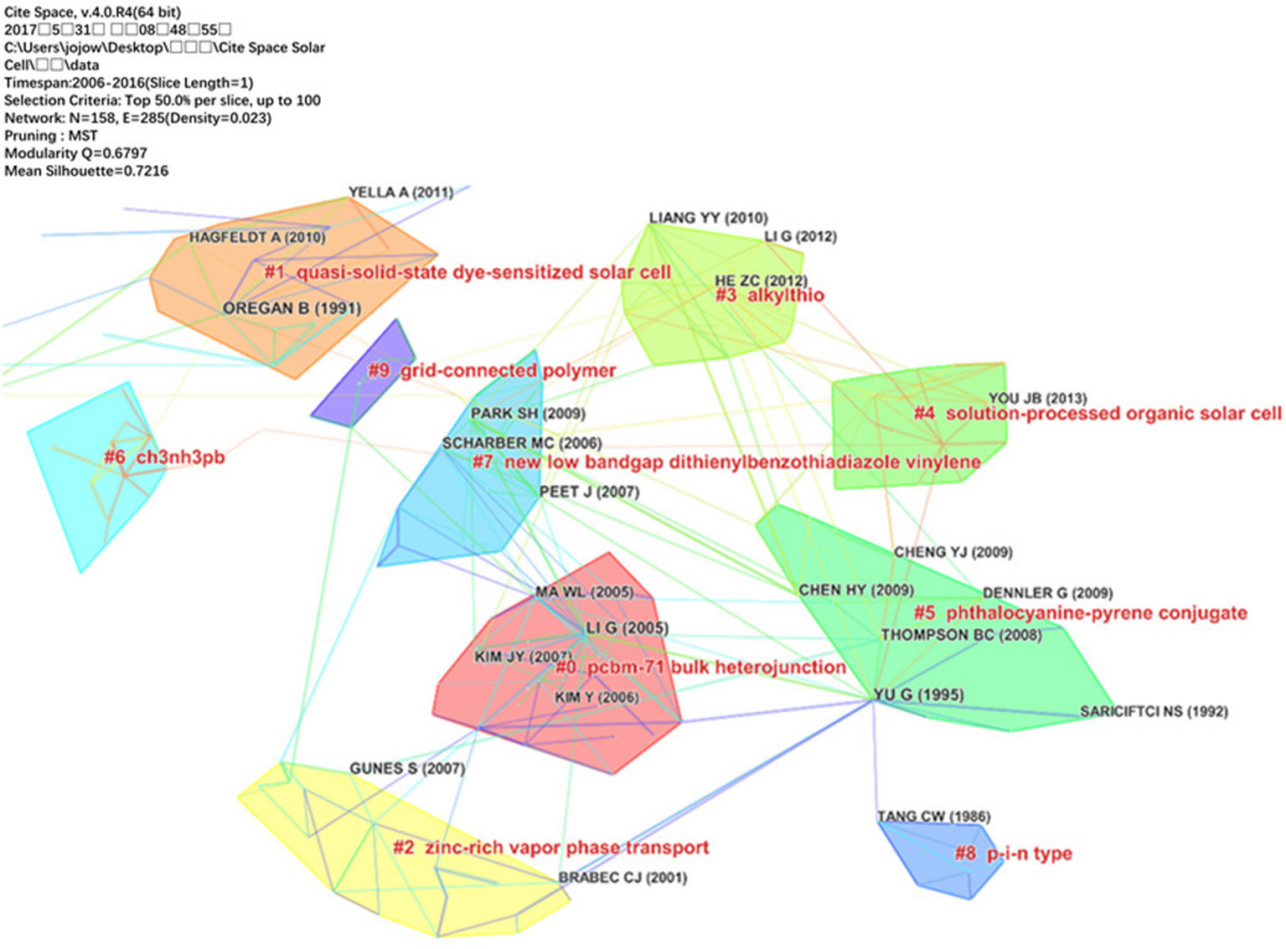
Clusters visualization based on a document co-citation network of 2006–2016.

A total set of 5,695 articles were visualized and analyzed using CiteSpace with a timespan from 2006 to 2016 and a time slice of 1 was chosen for the analysis. The selection criteria was the top 50 most cited or occurred items from each slice, and their document co-citation network pruned by MST was generated as shown in Figure 4. As a result, 158 unique nodes, 285 links and 10 main clusters were generated with a modularity Q of 0.6797 and a means silhouette of 0.7216. These nodes and links represent cited references and co-citation relationships from the collected articles, respectively. The link colors correspond directly to time slice which means that the cold colors represent the early years and the warm ones represent the near years. For example, purple links describe articles that were co-cited in 2006, and the most recent co-citation relationships are visualized as yellow or orange links. The modularity Q and the mean silhouette are two indicators to evaluate the clusters. Q > 0.3 means that the network is significant and the silhouette >0.5 means that the clustering result is rational.

Table 2 presents the top 10 cited references in OPV. Nodes with high betweenness can be considered as pivotal points that provide important bridging connections between two research interests. When ranked by betweenness centrality, the first is a paper published by Yu et al. (1995), which improved the carrier collection efficiency and energy conversion efficiency of polymer photovoltaic cells by blending of the poly (2-methoxy-5-(2′-ethyl-hexyloxy)-1,4-phenylene vinylene) (MEH-PPV) with C_60_ derivative and put forward the concept of network of internal donor-acceptor heterojunctions. The second is Li et al. (2005), which achieved a highest power conversion efficiency of 4.4% based on the polymer P3HT at that time by simple solution processing method with low cost. The other papers focus on improving the power conversion efficiency of the OPV device by diverse methods and study on the mechanism more and more deeply. For example, Ma et al. (2005) improved the device performance by thermal annealing to change the nanoscale morphology of bulk heterojunction material. Park et al. (2009) fabricated the solar cells based on poly[N-900-hepta-decanyl-2,7-carbazole-alt-5,5-(40,70-di-2-thienyl-20,10,30-benzothiadiazole) (PCDTBT) and the internal quantum efficiency is close to 100%, implying that essentially every absorbed photon results in a separated pair of charge carriers and all photogenerated carriers are collected at the electrodes. Scharber et al. (2006) based on the existed findings to derive a relation between energy-conversion efficiency of a bulk-heterojunction solar cell, bandgap, and the LUMO level of the donor, then proposed a model to guide the material selection and material development for bulk-heterojunction solar cells. He et al. (2012) demonstrated highly efficient polymer solar cells with a certified efficiency of 9.2% using an inverted structure based on polymer thieno[3,4-b]thiophene/benzodithiophene (PTB7), which simultaneously offered ohmic contact for photogenerated charge-carrier collection and allowed optimum photon harvest in the device. While there are other article papers with high centrality are valued to be mentioned, for example, “Aggregation and morphology control enables multiple cases of high-efficiency polymer solar cells” published by Liu et al. (2014) with betweenness centrality of 0.13. They controlled the morphology by temperature-dependent aggregation behavior of donor polymers, poly[(5,6-difluoro-2,1,3-benzothiadiazol-4,7-diyl)-alt-(3,3‴-di(2-octyldodecyl)-2,2′;5′,2″;5″,2‴-quaterthiophen-5,5‴-diyl)] (PffBT4T-2OD), (poly[(2,1,3-benzothiadiazol-4,7-diyl)-alt-(4′,3″-difluoro-3,3‴-di(2-octyldodecyl)-2,2′;5′,2″;5″,3‴-quaterthiophen-5,3‴-diyl)] (PBTff4T-2OD), poly[(naphtho[1,2-c:5,6-c′]bis[1,2,5] thiadiazol-5,1′-diyl)-alt-(3,3‴-di(2-octyldodecyl)-2,2′;5′,2″;5″,2‴-quaterthiophen-5,5‴-diyl)] (PNT4T-2OD) and yielded high-performance thick-film polymer solar cells with efficiency exceeding 10%. This work is meaningful for both materials synthetic advances and device performance improvement. In sum, these articles mentioned above showed the improvement in OPV performance from different aspects.

**Table 2:** Top 10 most cited articles in OPV field.

**Citation counts**	**Tile**	**Author**	**Year**	**Betweenness centrality**	**Journal**	**Cluster #**
3,871	Polymer photovoltaic cells: Enhanced efficiencies via a network of internal donor-acceptor heterojunctions	G. Yu et al.	1995	0.38	Science	5
3,661	A low-cost, high-efficiency solar cell based on dye-sensitized colloidal TiO_2_ films	B. Oregan et al.	1991	0.07	Nature	1
3,326	High-efficiency solution processable polymer photovoltaic cells by self-organization of polymer blends	G. Li et al.	2005	0.35	Nat. Mater.	0
2,970	Thermally Stable, Efficient Polymer Solar Cells with Nanoscale Control of the Interpenetrating Network Morphology	W. L. Ma et al.	2005	0.16	Adv. Funct. Mater.	0
2,717	Conjugated Polymer-Based Organic Solar Cells	S. Gunes et al.	2007	0.01	Chem. Rev.	2
2,422	Bulk heterojunction solar cells with internal quantum efficiency approaching 100%	S. H. Park et al.	2009	0.1	Nat. Photonics	7
2,346	Design Rules for Donors in Bulk-Heterojunction Solar Cells—Toward 10% Energy-Conversion Efficiency	M. C. Scharber et al.	2006	0.2	Adv. Mater.	7
2,222	Polymer-fullerene composite solar cells	B. C. Thompson et al.	2008	0.06	Angew. Chem. Int. Edit.	5
2,173	For the Bright Future—Bulk Heterojunction Polymer Solar Cells with Power Conversion Efficiency of 7.4%	Y. Y. Liang et al.	2010	0.04	Adv. Mater.	3
2,088	Enhanced power-conversion efficiency in polymer solar cells using an inverted device structure	Z. C. He et al.	2012	0.25	Nat. Photonics	3

Research patterns and emerging trends in the knowledge system in terms of key clusters of articles are explored. As shown in Figure 4, there are 10 co-citation clusters in the network and these clusters are labeled by index terms from their own citers. To characterize the nature of a cluster, CiteSpace can extract noun phrases from the titles of articles that cited the cluster based on three specialized metrics—TFIDF, log-likelihood tests (LLR) and mutual information tests (MI). LLR usually gives the best result in terms of the uniqueness and coverage of themes associated with a cluster. The detailed informations of the 10 clusters are summarized in Table 3.

**Table 3:** Top-ranked clusters in OPV field.

**ClusterID**	**Size**	**Silhouette**	**Label (TFIDF)**	**Label (LLR)**	**Label (MI)**	**Mean (cite year)**
#0	28	0.784	pcbm-71 bulk heterojunction	Dye-sensitized solar cell	6-phenyl c61 butyric acid methyl ester blend	2004
#1	21	0.984	Quasi-solid-state dye-sensitized solar cell	Dye-sensitized solar cell	Containing fluorene	2002
#2	18	0.768	Zinc-rich vapor phase transport	Perovskite solar cell	Direct application	2003
#3	16	0.792	Alkylthio	Solar cell	Graphene	2011
#4	16	0.841	Solution-processed organic solar cell	Small molecule	Acceptor interface	2013
#5	16	0.761	Phthalocyanine-pyrene conjugate	Synthesis	Absorbing small molecule	2003
#6	14	0.947	ch3nh3pb	Stable perovskite solar cell	Evolution	2011
#7	14	0.827	New low bandgap dithienylbenzothiadiazole vinylene	Synthesis	Crystallinity	2005
#8	7	0.981	p-i-n type	Organic photovoltaic cell	Flexible substrate	2000
#9	6	0.974	Grid-connected polymer	Manufacture	Flexible substrate	2009

The values of the silhouettes for each cluster are greater than 0.5, suggesting reliable and meaningful results. As shown in Figure 4, “pcbm-71 bulk heterojunction” is the largest cluster (#0) consisting 28 members. The most active citers in this cluster is Brunetti et al. (2010), “Organic electronics from perylene to organic photovoltaics: painting a brief history with a broad brush.” This paper reviewed the correlation between the performance of the device and the active layer composites and analyzed the motivations behind specific bulk-heterojunction designs in polymer solar cells. This paper reflected the researchers interests in cluster #0 generally. The second largest cluster (#1) in this knowledge domain, “quasi-solid-state dye-sensitized solar cell,” has 21 member articles and an average publication year of 2002. The most active citers to this cluster is Chen et al. (2010), “photophysical studies of dipolar organic dyes that feature a 1,3-cyclohexadiene conjugated linkage: the implication of a twisted intramolecular charge-transfer state on the efficiency of dye-sensitized solar cells,” which focuses on the dye-sensitized solar cells (DSSCs). The third largest cluster (#2) is “zinc-rich vapor phase transport” which has 18 members and an average publication year of 2003. The most active citers in this cluster is Canli et al. (2010), “chiral (s)-5-octyloxy-2-[{4-(2-methylbuthoxy)-phenylimino}-methyl]-phenol liquid crystalline compound as additive into polymer solar cells.” They found that the charge carrier mobility increased significantly in the devices with liquid crystals additions.

There are other clusters in Figure 4. worth mentioning. For example, cluster #3 has the top ranked burst article published by He et al. (2012) among all clusters, with bursts of 290.34, which represent the active area and emerging trend (Kleinberg, 2002). This work constructed inverted device structure and boosted in efficiency drastically. This discovery could be used in various material systems, and also open up new opportunities to improve performance of polymer solar cells. The second ranked burst article published by You et al. (2013) with bursts of 290.34 in cluster #4. This work first certified polymer solar cell efficiency over 10% by using a tandem structure based on their low bandgap polymer poly[2,7-(5,5-bis-(3,7-dimethyloctyl)-5H-dithieno[3,2-b:2′,3′-d]pyran)-alt-4,7-(5,6-difluoro-2,1,3-benzothia diazole)]. The third ranked burst article in cluster #6 by Burschka et al. (2013) with bursts of 220.72, which provide a route to fabricate solution-processed perovskite-sensitized solar cells. In summary, from the top three ranked burst articles it can be concluded that the inverted device structure and tandem solar cells are the emerging trend in OPV.

### Emerging trends

Significant increases of research interests in the OPV field are highlighted by publications with citation bursts. Table 4 shows the top 30 references among a total of 116 references with the strongest citation bursts during the period between 2006 and 2016. As shown in Table 4, the first 3 ranked references all started to burst in 2014 which represented the emerging trends of OPV and we have discussed in detail in front part. While some representative references started to burst from different years among the 116 references, which reflect emerging trends in different period of time and give expression to the development track of OPV, are listed in Table 5.

**Table 4:** Top 30 references with strongest citation bursts.

**References**	**Year**	**Strength**	**Begin**	**End**	**2006–2016**
He ZC, 2012, NAT PHOTONICS, V6, P591	2012	290.3443	2014	2016	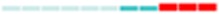
YOU JB, 2013, NAT COMMUN, V4, P, doi: 10.1038/2411	2013	267.6309	2014	2016	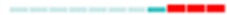
BURSCHKA J, 2013, NATURE, V499, P316	2013	220.7215	2014	2016	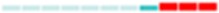
BRABEC CJ, 2001, ADV FUNCT MATER, V11, P15	2001	191.6483	2006	2009	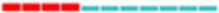
LIU MZ, 2013, NATURE, V501, P395	2013	182.501	2014	2016	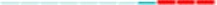
SHAHEEN SE, 2001, APPL PHYS LETT, V78, P841	2001	154.5118	2006	2010	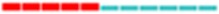
KIM JY, 2007, SCIENCE, V317, P222	2007	139.1624	2008	2011	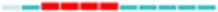
CHEN HY, 2009, NAT PHOTONICS, V3, P649	2009	138.6255	2011	2012	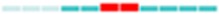
MA WL, 2005, ADV FUNCT MATER, V15, P1617	2005	138.0514	2007	2010	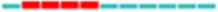
LI G, 2012, NAT PHOTONICS, V6, P153	2012	125.2167	2013	2016	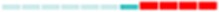
PADINGER F, 2003, ADV FUNCT MATER, V13, P85	2003	122.5108	2006	2009	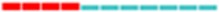
DOU LT, 2012, NAT PHOTONICS, V6, P180	2012	119.2042	2013	2014	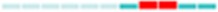
YELLA A, 2011, SCIENCE, V334, P629	2011	114.7987	2013	2016	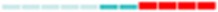
COAKLEY KM, 2004, CHEM MATER, V16, P4533	2004	114.5338	2006	2010	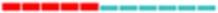
REYES-REYES M, 2005, APPL PHYS LETT, V87	2005	112.2608	2006	2010	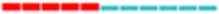
SARICIFTCI NS, 1992, SCIENCE, V258, P1474	1992	108.1084	2006	2010	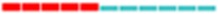
CHU TY, 2011, J AM CHEM SOC, V133, P4250	2011	103.3506	2012	2013	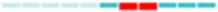
PARK SH, 2009, NAT PHOTONICS, V3, P297	2009	96.3446	2010	2011	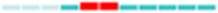
DOU LT, 2013, ADV MATER, V25, P6642	2013	90.144	2014	2016	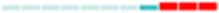
LI G, 2005, NAT MATER, V4, P864	2005	89.3459	2007	2010	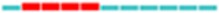
HE ZC, 2011, ADV MATER, V23, P4636	2011	87.5289	2012	2014	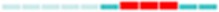
HUYNH WU, 2002, SCIENCE, V295, P2425	2002	86.4289	2006	2009	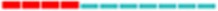
LI YF, 2012, ACCOUNTS CHEM RES, V45, P723	2012	86.0756	2013	2016	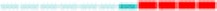
PEUMANS P, 2003, J APPL PHYS, V93, P3693	2003	85.1588	2006	2009	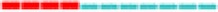
ZHOU JY, 2013, J AM CHEM SOC, V135, P8484	2013	84.1867	2014	2016	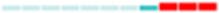
GUO XG, 2013, NAT PHOTONICS, V7, P825	2013	81.8066	2014	2016	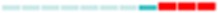
HALLS JJM, 1995, NATURE, V376, P498	1995	79.2972	2006	2009	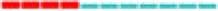
CABANETOS C, 2013, J AM CHEM SOC, V135, P4656	2013	77.1019	2014	2016	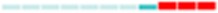
LIN YZ, 2012, CHEM SOC REV, V41, P4245	2012	72.4234	2014	2016	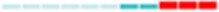
SPANGGAARD H, 2004, SOL ENERG MAT SOL C, V83, P125	2004	71.336	2006	2009	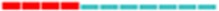

**Table 5:** Representative references ranked by the beginning time of burst.

**References**	**Title**	**Burst**	**Burst duration**	**Range (2006–2016)**
J. C. Brabec et al., 2001, *Adv. Func. Mater*. (Brabec et al., 2001)	Plastic Solar Cells	191.6483	2006–2009	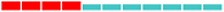
J. Y. Kim et al., 2007, *Science* (Kim et al., 2007)	Efficient Tandem Polymer Solar Cells Fabricated by All-Solution Processing	139.1624	2008–2011	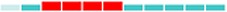
H. Y. Chen et al., 2009, *Nat. Photonics* (Chen et al., 2009)	Polymer solar cells with enhanced open-circuit voltage and efficiency	138.6255	2011–2012	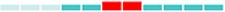
T. Y. Chu et al., 2011, *J. Am. Chem. Soc*. (Chu et al., 2011)	Bulk heterojunction solar cells using thieno[3,4-c]pyrrole-4,6-dione and dithieno[3,2-b:2′,3′-d]silole copolymer with a power conversion efficiency of 7.3%	103.3506	2012–2013	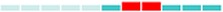
L. T. Dou et al., 2012, *Nat. Photonics* (Dou et al., 2012)	Tandem polymer solar cells featuring a spectrally matched low-bandgap polymer	119.2042	2013–2014	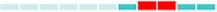
Z. C. He et al., 2012, *Nat. Photonics* (He et al., 2012)	Enhanced power-conversion efficiency in polymer solar cells using an inverted device structure	290.3443	2014–2016	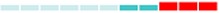
J. B. You et al., 2013, *Nat. Commun*. (You et al., 2013)	A polymer tandem solar cell with 10.6% power conversion efficiency	267.6309	2014–2016	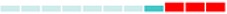
J. Burschka et al., 2013, *Nature* (Burschka et al., 2013)	Sequential deposition as a route to high-performance perovskite-sensitized solar cells	220.7215	2014–2016	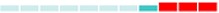
M. Z. Liu et al, 2013 *Nature* (Liu et al., 2013)	Efficient planar heterojunction perovskite solar cells by vapor deposition	182.501	2014–2016	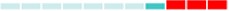
L. T. Dou et al., 2013, *Adv. Mater*. (Dou et al., 2013)	25th anniversary article: a decade of organic/polymeric photovoltaic research	90.144	2014–2016	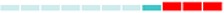
J. Y. Zhou et al., 2013, *J. Am. Soc. Chem*. (Zhou et al., 2013)	Solution-processed and high-performance organic solar cells using small molecules with a benzodithiophene unit	81.1867	2014–2016	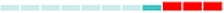
X. G., Guo et al., 2013, *Nat. Photonics* (Guo et al., 2013)	Polymer solar cells with enhanced fill factors	81.8066	2014–2016	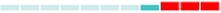
C. C. Cabanetos et al., 2013, *J. Am. Soc. Chem*. (Cabanetos et al., 2013)	Linear side chains in benzo[1,2-b:4,5-b']dithiophene-thieno[3,4-c]pyrrole-4,6-dione polymers direct self-assembly and solar cell performance	77.1019	2014–2016	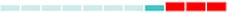
Y. H. Zhou et al., 2012, *Science* (Zhou et al., 2012)	A Universal Method to Produce Low-Work Function Electrodes for Organic Electronics	71.1696	2014–2016	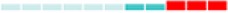

Table 5 shows the representative references for three groups by the beginning time of burst which can reflect the development history of OPV. The earliest references with the strongest citation bursts are published by Brabec et al. (2001) with burst duration from 2006 to 2009. It is one of the earliest reviews about polymer solar cells which introduced some basic concepts of OPV such as bulk heterojunction, device architectures, the donor conjugated polymers, and performance improving strategy. Subsequently, Kim et al. (2007) successfully demonstrated the application of polymer-based bulk heterojunction tandem cells, by using poly[2,6-(4,4-bis-(2-ethylhexyl)-4H-cyclopenta[2,1-b;3,4-b'] dithiophene)-alt-4,7-(2,1,3-benzothiadiazole)](PCPDTBT) and poly(3-hexylthiophene)(P3HT) as the active layer respectively and with each layer processed from solution. The burst last 4 years from 2008 till 2011.

Then by following the development of bulk heterojunction structure and the study of the relationship between the open-circuit voltage and energy levels of donor/acceptor in bulk heterojunction polymer solar cells, Chen et al. (2009) tuned the open-circuit voltage of the device based on polymer PBDTTT by introducing different functional groups on the backbone of the polymer chain. This work provided a new material design strategy for constructing high performance devices and this reference burst 2 years from 2011 to 2012. The reference burst from 2012 to 2013 published by Chu et al. (2011) reported a new alternating copolymer of dithienosilole and thienopyrrole-4,6-dione (PDTSTPD), which exhibited a power conversion efficiency of 7.3% on the photovoltaic devices when blending with PC_71_BM. L. T. Dou et al. introduced the new low-bandgap conjugated polymer PBDTT-DPP to construct the tandem solar cell and achievement a high efficiency of 8.7% in 2012 and this paper burst from 2013 to 2014. Therefore, from the representative reference with the strongest citation burst duration in the period from 2011 to 2014, we can conclude that during this time the research hotspot in OPV was the preparation of new conjugated polymer materials.

As shown in Table 5, the nearest burst duration is from 2014 to 2016 which represent the emerging trends of OPV. The first is published by He et al. (2012). They constructed an inverted device and improved the performance of polymer solar cells significantly which is a meaningful work because it can be applied in many material systems. You et al. (2013) reported the tandem structure solar cells with an efficiency higher than 10% for the first time. In 2003, Burschka et al. (2013) reported a route to high-performance perovskite-sensitized solar cells which drive the research of perovskite-sensitized solar cells vastly. Well small molecular solar cell with some unique advantages is another important branch of OPV, but the performance of small molecular solar cells is relatively poor until Zhou et al. (2013) published the paper in 2013 with a burst of 81.1867. They designed and synthesized small molecules incorporating the advantages of both conventional polymers and small molecules synergistically which is meaningful for guiding the small molecules design. The reference with a burst of 77.1019 published by Cabanetos et al. (2013) studied the impacts of varying size and branching of solubilizing side chains in π-conjugated polymers to their self-assembling properties in thin-film devices. After that, Yan et al. (Liu et al., 2014) and Chen Z. et al. (2014) studied the impacts of side chains in conjugated polymer chains on the morphology of the polymer solar cell films. The optoelectronic devices with at least one low work function electron to inject or collect electrons from the organic semiconductors are required. Therefore, to modify the electrode of OPV devices with some interface materials is an important research topic. Zhou et al. (2012) modify the electrode with polymers containing simple aliphatic amine groups and reduce the work function of conductors including metals, transparent conductive metal oxides, conducting polymers, and graphene substantially. This reference published in 2012 begin to burst from 2014. So, from analysis of the representative reference with the strongest citation burst duration from 2014 to 2016, we can conclude that the emerging trends of OPV are mainly about the device structures of solution processing polymer solar cells such as the inverted solar cells and the tandem ones, small molecule solar cells, side chains in π-conjugated polymers and the interface modification of device electrodes.

**Figure 5:**
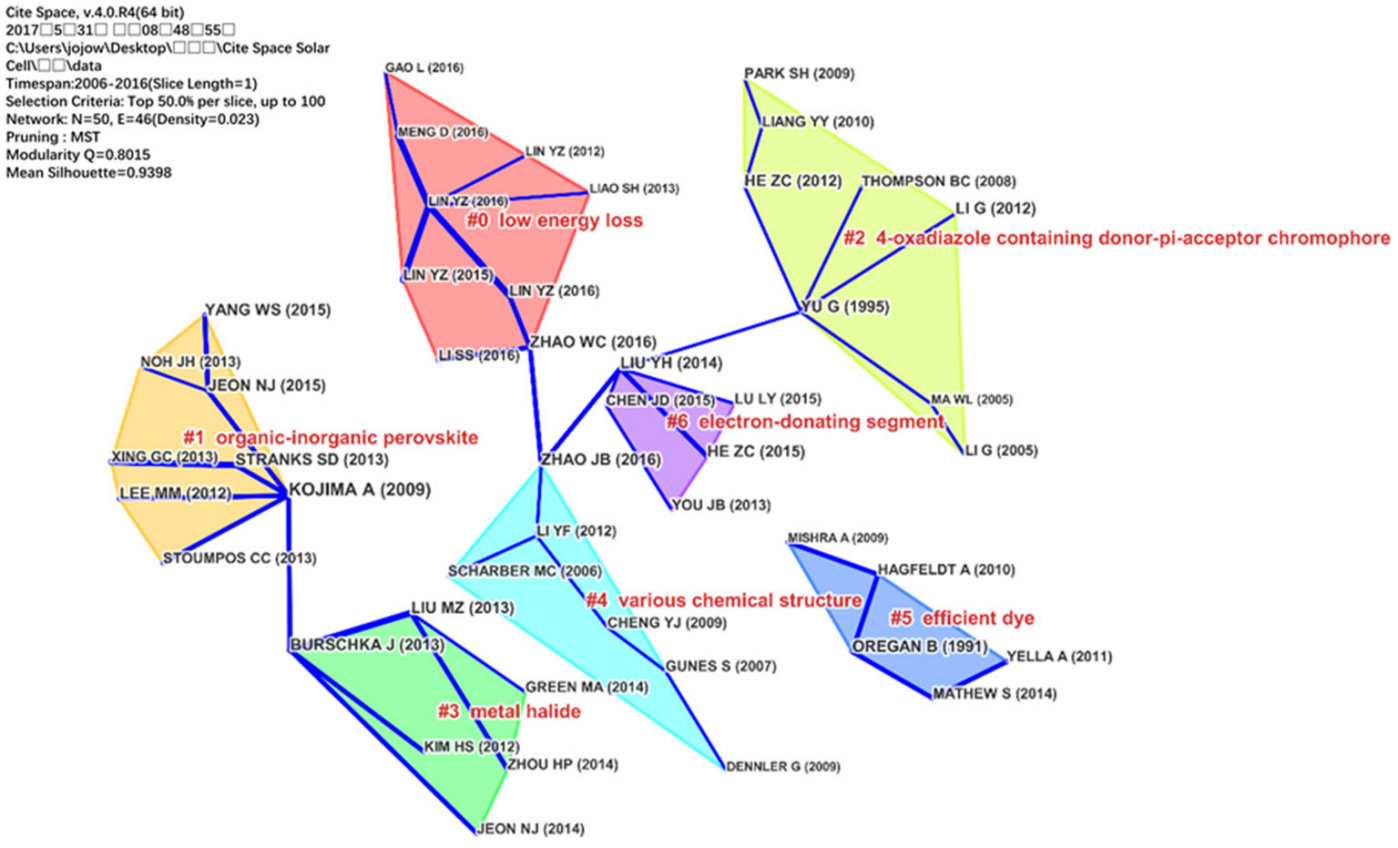
Clusters visualization based on a document co-citation network of 2017.

To further confirm the developments of OPV, the papers published in 2017 were analyzed by CiteSpace. As shown in Figure 5, there are 7 co-citation clusters in the network and these clusters are labeled by index terms from their own citers. “low energy loss” is the largest cluster (#0) consisting 9 members. The most active citers in this cluster is Li S. X. et al. (2017) “molecular electron acceptors for efficient fullerene-free organic solar cells.” This paper reviewed the designing rules as well as perspectives for the development of non-fullerene acceptors. This paper reflected the researchers interests in cluster #0 generally. The second largest cluster (#1) in this knowledge domain, “organic-inorganic perovskite,” has 8 members. The most active citers to this cluster is Bakr et al. (2017) “advances in hole transport materials engineering for stable and efficient perovskite solar cells,” which focus on the hole transport materials used in perovskite solar cells. As shown in Figure 5, cluster #1 and cluster #3 are mainly about perovskite solar cell and cluster #5 is about DSSC, and the other clusters are about OPV. It is clearly that there are no links between perovskite clusters and OPV clusters, so as to DSSC cluster.

### Co-citation analysis of all-polymer solar cells

**Figure 6:**
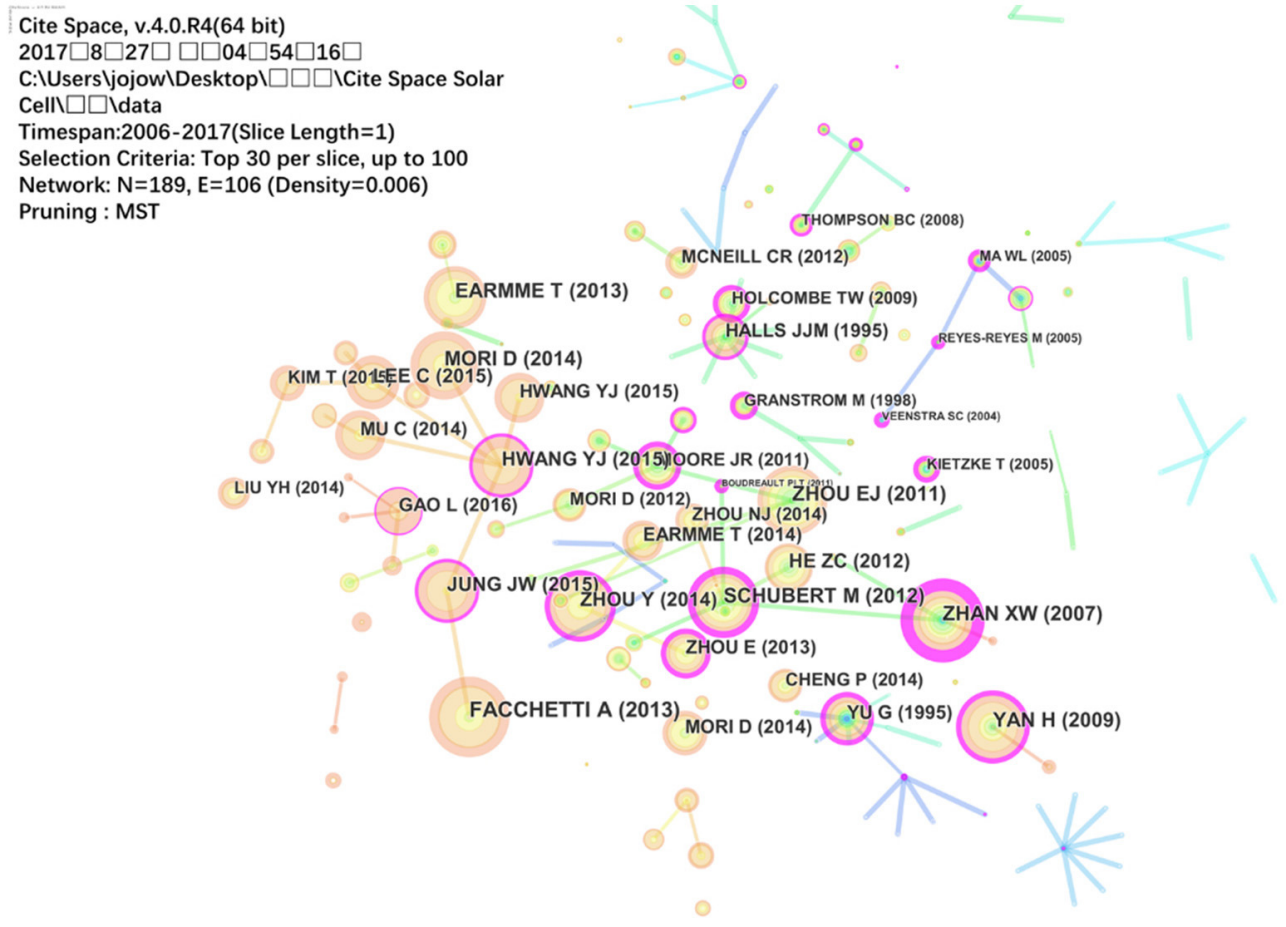
Visualization based on a document co-citation network of all-polymer solar cells.

As previous analysis, it can be found that the development of polymer solar cells with no-fullerene acceptors is an emerging trend in OPV. Therefore, all-polymer solar cells, consisting of polymer donors and polymer acceptors, have recently been studied extensively. Then we used “all polymer solar cells” as the index word in title for search and the resulted article document type records were exported to CiteSpace for analyzing. As shown in Figure 6, the size and purple color stand for the centrality and importance of the nodes. The top ranked item by centrality is Zhan et al. (2007) with centrality of 1.39. They reported the perylene diimide (PDI) based n-type polymer Poly{[N,N'-bis(2-decyl-tetradecyl)-3,4,9,10-perylene diimide-1,7-diyl]-alt-(dithieno[3,2-b:2′,3′-d]thiophene-2,6-diyl)} which can be used as the acceptor of polymer solar cells (Zhan et al., 2007). The second one is Schubert et al. (2012) with centrality of 0.64. They reported the naphthalenediimide (NDI)-based copolymers as acceptors and regioregular P3HT as the donor and PCE >1% is achieved for rylene-based polymer acceptors for the first time (Schubert et al., 2012). The third is Yan et al. (2009) with centrality of 0.54. NDI-based polymer poly{[N, N9-bis(2-octyldodecyl)-naphthalene-1,4,5,8-bis(dicarboximide)-2,6-diyl]-alt-5,59-(2,29-bithiophene)}, (P(NDI2OD-T2) was synthesized and used to fabricate the printed transistor with a high electron mobility (Yan et al., 2009). Other important nodes were also presented in Figure 6. A serious of n-type copolymers based on PDI and NDI units were synthesized and used as the acceptor materials of all-polymer solar cell, because the unique characters of the PDI or NDI, including the high electron affinity of the rylene diimide core caused by two strong electron-withdrawing diimide groups and a highly extended π-conjugated structure that produces strong intermolecular π-π interactions. Based on the contributions of the achievements shown in Figure 6, the PCE values of all-polymer solar cells have risen to 8% (Kang et al., 2016).

## Conclusions

In conclusion, the co-citation analysis and visualized network of the reference about OPV technology were calculated by CiteSpace at first. Then the key clusters of articles and identified research patterns and emerging trends in the literature were explored based on the results of CiteSpace (Chen et al., 2009). By studying the key references explored by software in the clusters, it can be known that the main knowledge domains are synthesis of novel molecules, the film morphology control, the device mechanisms and constructing new device architectures. From the detected burst of citations, it can be concluded that the inverted device structure and tandem solar cells are the emerging trend in OPV and perovskite solar cell is a new important branch of organic solar cells. By analyzing the articles published in 2017, it can be found that non-fullerene acceptors for high efficiency solar cells was an emerging trend in OPV.

Well due to the interdisciplinary characteristic of OPV, it is difficult to obtain an overall picture of the research field. But we have demonstrated a quantitative scientometric method to explore the advance of the collective knowledge of OPV by tapping into the references published in this field, which can help us to understand the discern patterns and trends in this field visually efficiently.

Compared with the reviews from domain experts, the analyses based on CiteSpace in this paper could be controversial and somewhat shallow. Drawbacks existed in CiteSpace, for examples, as shown in Figure 2, the first author and corresponding author cannot be distinguished clearly. Some co-keywords shown in Figure 3 are similar which should be merged in the same circle, such as “efficiency” and “high efficiency,” “performance,” and “high performance.” While it is believed that as the efforts of the research group of CiteSpace, this software will be updated to overcome this drawbacks and present more accurate and deep knowledge domain in the future.

## Author contributions

FX: Conceived and designed the analysis. Collected the data. Contributed data or analysis tools. CL: Conceived and designed the analysis. JS: Conceived and designed the analysis. Collected the data. Wrote the paper. LZ: Revise the paper.

### Conflict of interest statement

The authors declare that the research was conducted in the absence of any commercial or financial relationships that could be construed as a potential conflict of interest.
